# Incidence and risk factors of hyperglycemic emergencies in adults with diabetes in the public hospitals in Harari region, Eastern Ethiopia

**DOI:** 10.1371/journal.pgph.0007345

**Published:** 2026-09-25

**Authors:** Birhanu Shegene, Abdi Birhanu, Alemayehu Tesfaye, Abera Cheru, Nano Belema, Kasiye Shiferaw

**Affiliations:** 1 Department of Epidemiology and Biostatistics, School of Public Health, College of Health and Medical Sciences, Haramaya University, Harar, Ethiopia; 2 Department of Epidemiology and Biostatistics, School of Medicine, College of Health and Medical Sciences, Haramaya University, Harar, Ethiopia; 3 School of Midwifery, College of Health and Medical Sciences, Haramaya University, Harar, Ethiopia; 4 School of Environmental Health Science, College of Health and Medical Sciences, Haramaya University, Harar, Ethiopia; 5 Department of Midwifery, College of Medicine and Health Sciences, Dire Dawa University, Dire Dawa, Ethiopia; PLOS: Public Library of Science, UNITED STATES OF AMERICA

## Abstract

Hyperglycemic emergencies are the most common life-threatening acute metabolic complications of diabetes mellitus. The number of hyperglycemic emergency admissions over the past decade has increased worldwide, with recent data reporting a 55% increase in the rate of diabetic ketoacidosis hospitalizations. Therefore, this study aimed to determine the incidence and predictors of hyperglycemic emergencies among adult diabetic patients at public hospitals in the Harari Region, Eastern Ethiopia. A retrospective cohort study was conducted among 496 adults with diabetes. Data were collected using the Kobo toolbox and analyzed using STATA. The Kaplan-Meier curve and log-rank test were used to estimate the survival probability and statistically test survival between different categories, respectively. Predictors of hyperglycemic emergencies were determined using the Cox proportional hazard regression model. Hazard ratios (HR) with their respective 95% confidence interval (CI) and p-value were computed. Of the 472 patients included in the study, 34.11% (95% CI: 29.96%-38.52%) developed hyperglycemic emergencies. The incidence rate of hyperglycemic emergencies was 18.6 per 100 person-years observation (95%CI: 15.9-21.7). The median hyperglycemic emergency-free survival time was 51.06 months (95% CI: 45.14-58.56). Being a rural area residents (Adjusted Hazard Ratio(AHR): 1.52, 95% CI: 1.07,2.16), not having community health insurance (AHR: 1.63, 95% CI: 1.14-2.34), experiencing acute recent illness (AHR: 2.15, 95% CI: 1.51,3.07), having type 1 diabetes mellitus (AHR: 1.97, 95% CI: 1.23,3.15), having poorly controlled blood glucose (AHR: 3.06, 95% CI: 2.01,4.66), non-adherence with diabetes medication (AHR: 2.25, 95% CI: 1.55,3.26), being obese (AHR: 3.75, 95% CI: 1.84,7.64) and overweight (AHR: 2.56, 95% CI: 1.72,3.81) were factors significantly associated with hyperglycemic emergencies. The findings demonstrated that the incidence of hyperglycemic emergencies was high. Hence, special emphasis should be placed on follow-up care for diabetic patients with identified predictors to reduce the occurrence of these emergencies.

## Introduction

Diabetic ketoacidosis (DKA) and hyperosmolar hyperglycemic state (HHS) are collectively referred to as hyperglycemic emergencies (HGEs). These are the two most common life-threatening acute metabolic complications of diabetes mellitus (DM) and are associated with uncontrolled type 1 and 2 diabetes [1]. DKA is characterized by a combination of hyperglycemia (serum glucose > 250 mg/dL), acidosis (arterial blood pH < 7.3 and bicarbonate < 15 mEq/L), and ketosis (ketonuria or ketonemia) [2]. It frequently occurs in young patients with type 1 diabetes, and the rate of DKA at presentation ranges from 15 to 70% [1]. According to current international guidelines, HHS is defined as severe hyperglycemia, usually > 600 mg/dL, hyperosmolality (osmolality greater than 320 mOsm/L), and dehydration without ketoacidosis [3]. HHS is more common among older patients with type 2 diabetes and occurs in approximately 2% of adolescents at the time of presentation. However, both DKA and HHS can occur in either type of diabetes mellitus [1].

Most cases of HGE occur in people who have previously been diagnosed with diabetes. Some patients develop these complications due to inadequate glycemic control during the treatment process [4]. Therefore, it is estimated that 50% of hospitalizations could be prevented through improved follow-up care and better adherence to self-care practices [5]. The World Health Organization reports that 1.5 million deaths are directly attributable to diabetes each year [6]. HGEs are a leading cause of diabetes-related morbidity, mortality, and healthcare costs in adults with diabetes [7]. The burden and consequences of diabetes have risen dramatically over recent years [8]. As a result, HGEs remain a significant health and socioeconomic problem among adults with diabetes mellitus [9,10].

In developing countries, the mortality rate for HGEs is approximately 20%, with case fatality rates of 18% for DKA and 35% for HHS [1]. In Africa, mortality rates associated with HGEs vary widely, ranging from 7.5% in South Africa to 34% in Nigeria [11]. Various studies have shown that mortality rates are 2–5% for DKA and 10–20% for HHS in other regions [12,13]. Premature mortality is particularly high in Sub-Saharan Africa, including Ethiopia [8]. In Eastern Ethiopia, the mortality rate for HGEs was 16.5%, with 21.4% for HHS and 11.1% for DKA [14].

Studies conducted in various parts of the world have reported varying incidence rates of HGEs. Incidence rates of DKA range from 0 to 56 cases per 1000 person-years, with one outlier study from China reporting an exceptionally high incidence rate of 263 cases per 1000 person-years. Conversely, the lowest incidence rates (0 cases per 1000 person-years) were reported in Israel and North America [15]. According to a report by the American Diabetes Association, the exact global incidence of HHS is unknown, but it is estimated to account for less than 1% of hospital admissions in patients with diabetes [16]. In contrast, a study conducted at Bahir Dar public hospitals in Ethiopia reported an incidence rate of 2.1 cases of HHS per 100 person-years, with rates of 0.9 and 2.4 cases per 100 person-years among patients with T1DM and T2DM, respectively [17]. Across Africa, more than 12% of hospitalizations for diabetes are attributed to hyperglycemic crises [18], with hospitalization rates ranging from 26% in South Africa to 40% in Nigeria [11]. Another study in Ethiopia reported an overall incidence rate of 14.6 cases per 100 person-years observation [17].

The incidence of HGEs is influenced by socio-demographic factors, clinical characteristics, and treatment-related factors, and these predictors often vary across different studies. Hyperglycemic crises commonly arise secondary to infections (accounting for 30–50% of cases), poor adherence to diabetes medications, intercurrent diseases, psychological stress, alcohol/drug abuse, trauma, and new-onset type 1 diabetes [12,18]. In resource-limited countries like Ethiopia, identifying the incidence and predictors of HGEs among patients with diabetes is crucial for developing effective prevention and management strategies. This, in turn, could support efforts to achieve the Sustainable Development Goal (SDG) aimed at reducing premature mortality from chronic conditions like diabetes.

Despite an increasing number of studies reporting the incidence and mortality of HGEs in developing countries, important knowledge gaps remain. Most prior research has focused on the prevalence of HGEs and has highlighted the need for further research on their incidence [19]. To the best of the researcher’s knowledge, there are a few studies that have been conducted on the incidence of HGEs among adults with diabetes in Ethiopia [17,20]. This previous research did not account for recurrent HGEs and lacked a comprehensive examination of critical variables such as body mass index, blood pressure, urine ketone/glucose levels, and family history of diabetes mellitus. Given the growing impact of HGEs, there is an information gap related to the incidence and predictors of HGEs, especially in the study area. Therefore, this study aimed to determine the survival, incidence, and predictors of HGEs among adult diabetic patients at public hospitals in the Harari Region, Eastern Ethiopia.

## Materials and methods

### Ethics statement

The studies involving humans were approved by the Institutional Health Research Ethics Review Committee of the Haramaya University College of Health and Medical Sciences (IHRERC/149/2024). The studies were conducted in accordance with the local legislation and institutional requirements. The ethics committee/institutional review board waived the requirement of written informed consent for participation from the participants or the participants’ legal guardians/next of kin due to the retrospective nature of the study.

### Study design, setting and period

A facility-based retrospective cohort study design was conducted among adult patients with diabetes on chronic follow-up for treatment from July 01, 2019 to June 30, 2024 and data were extracted from July 01–31, 2024, at public hospitals in the Harari region, which is situated in the Harari regional state, 526 kilometers from Ethiopia’s capital, Addis Ababa. It is estimated that there would be 283,000 population living in the Harari region, with a 1:1 male-to-female ratio [21]. There are two public, two private, one police, and one non-government hospital currently in the region that serve this population. In addition to those hospitals, there are 9 health centers, 29 private clinics, 26 health posts, and one regional laboratory to serve the community of the region. The study was conducted in two public hospitals located in the region: Hiwot Fana Comprehensive Specialized University Hospital (HFCSUH) and Jugal General Hospital. HFCSUH is a large teaching hospital with 210 beds, and it serves as a major referral center for about 5.8 million population in eastern Ethiopia [22]. The Jugal General Hospital has 95 beds, and it serves the population in Harar town. Currently, a total of around 1700 patients with DM visit these hospitals to receive their regular follow-up care (i.e., 914 in Hiwot Fana Comprehensive Specialized University Hospital and 786 in Jugal General Hospital) [23].

### Populations and eligibility

All adult patients with diabetes on chronic follow-up for treatment at public hospitals in the Harari region were considered as the source population. The study population included all adult patients with diabetes on chronic follow-up between July 01, 2019 and June 30, 2024, at public hospitals in the Harari region. All adult patients with diabetes enrolled in the chronic follow-up care at public hospitals in the Harari region, who were diagnosed between July 01, 2019 and June 30, 2024, were included in the study. However, Patients who developed HGEs at the time of their initial diabetes diagnosis and those who were transferred in from other facilities were excluded.

### Sample size and sampling procedures

The initial sample size of 451 was determined using the Schoenfeld formula via Stata software version 17, employing power analysis for the Cox proportional hazard model [24]. This was calculated by considering a 95% confidence interval, 80% power, 32.45% probability of an event, and an AHR of 1.63 for community health insurance from a previous study [17]. To account for a 10% anticipated withdrawal or incomplete records probability, the final target sample size was increased to 496.

All public hospitals in the Harari region, namely Hiwot Fana Comprehensive Specialized University Hospital and Jugal General Hospital, were selected. Prior to sample allocation, the number of newly diagnosed patients with diabetes who visited both hospitals for follow-up care within the study period was determined. A total of 2,028 adult patients with diabetes were identified, with 1,126 patients from Hiwot Fana Comprehensive Specialized University Hospital and 902 from Jugal General Hospital, during the period between July 1, 2019, and June 30, 2024.

The total sample size of 496 was proportionally allocated to the two hospitals based on the number of patients with diabetes receiving follow-up care at each hospital. Subsequently, a list of medical record numbers of adults diagnosed with DM was prepared using diabetic follow-up logbooks from both hospitals. Finally, the required number of participants was selected randomly using Excel-generated random numbers from the sampling frame (the list of diabetic patients enrolled from July 01, 2019 to June 30, 2024).

### Study variables

The incidence rate and time to develop HGEs were the outcome variables, while the independent variables included socio-demographic factors (age, sex, residence, community health insurance), clinical factors (body mass index (BMI), urine ketone/glucose, family history of DM, recent acute illness, type of DM, glycemic control, DM duration, presence of comorbidities, systolic blood pressure, diastolic blood pressure, presence of chronic diabetic complications), and treatment factors (DM follow-up frequency, type of drug used, duration of treatment, and medication non-adherence).

### Data collection instrument and procedure

The data was collected using a data extraction format adapted from various relevant literature [17,25–30]. The data extraction format consisted of five sections: general information, socio-demographic characteristics, clinical characteristics, treatment-related parameters, and the follow-up form. First, all patients with diabetes on chronic follow-up between July 01, 2019 and June 30, 2024, were identified from the DM registration book. Eligible adult patients were then randomly selected using Excel-generated random numbers, after individuals who developed HGEs during the first diagnosis of DM and transferred in were excluded. Data were collected from patients’ charts using a pretested data extraction format. Medical registration numbers were used to identify reviewed records. Data were collected by four BSc public health professionals who had experience in data collection, under the supervision of one trained BSc nurse who was working on chronic follow-up and one MPH public health professional.

### Data quality control

To maintain data quality, data collectors and supervisors received one-day of training focused on the data extraction system, data collection tools, and the study's objective. Before the actual data collection, a pretest was conducted at HFCSUH on 5% [25] of the sample size. Necessary modifications were made to the data extraction format. A week before the actual data collection, charts were cross-checked to ensure that the instruments were adequate, the checklist of tools was completed on time, and the data for the charts were complete. Close monitoring and supervision were carried out throughout the data collection period by both the principal investigator and the supervisors. Every day, the data collectors received the required feedback. The collected data were reviewed for completeness before analysis. Efforts were made to minimize misclassification bias by using uniform ascertainment criteria for clinical variables with more than one diagnostic criterion. A complete case analysis was used to handle missing data.

### Operational definitions

***Time scale:*** The survival time calculated in months based on the interval between the date of DM diagnosis and either the date of the first incident of HGEs or the date of censorship.

***Event:*** The occurrence of HGEs

***Censored:*** Adults diagnosed with DM who did not develop HGEs during the follow-up study (transfer out, died, lost to follow-up, and still not developed HGEs as the study ends)

***Incomplete records:*** Incomplete records are charts that lack complete information for the variables of the date of DM diagnosis, the date when HGE occurred, and follow-up history after diagnosis

**Hyperglycemic emergencies/crisis:** The occurrence of the first incident of DKA (random blood sugar (RBS) >250 mg/dl and urine ketone body >+2) or HHS (RBS > 600 mg/dl with minimal or absent urine ketone body), based on the clinical decisions of the physicians, and is confirmed from the patient’s medical records [31].

***Glycemic control:*** The average three-month fasting blood sugar (FBS) level ranges from 70 to 130 mg/dl or HgA1c of less than 7%, referred to as good glycemic control, and poor glycemic control is defined as an average FBS of > 130 or < 70 mg/dl or HgA1c of greater than 7% [32].

***Recent acute illness:*** Any patient who had an illness that occurred immediately before the outcome and whose disease is of rapid onset or has a short course [5].

***Medication non-adherence:*** Documentation indicating that the patient has stopped (discontinued) taking prescribed anti-diabetic medications [33].

***Adult***: Patients whose age is 18 years or older.

### Data processing and analysis

After data collection was complete, the collected data were exported from the Kobo toolbox to Microsoft Excel and then imported into STATA software version 17 for further data cleaning and analysis. Basic descriptive analyses were conducted, reporting mean and standard deviation or median and IQR for continuous variables, whereas frequency distribution and percentage were used for categorical variables. Each patient was followed until the occurrence of HGEs or any of the censored states, whichever occurred first. Survival analysis was employed as the appropriate statistical model to estimate the associations of HGEs incidence and its predictors. The HGE's free survival probability was calculated in months, using the time interval between DM diagnosis and the date of occurrence of HGE or censoring. The incidence rate of HGEs was computed. Life tables were used to estimate probabilities of survival after diagnosis of DM at different time intervals. Kaplan Meier survival curve and log-rank tests were performed to estimate the survival probability and to statistically compare survival between different categories of predictors, respectively.

The Schoenfeld residuals test for the proportional hazards assumption was conducted for individual predictors and global tests [24]. Each predictor had a P-value greater than 0.05, and the overall global test had a P-value of 0.252, indicating that the proportional hazard assumption was met and not violated. Plots were used to evaluate the predictors in the model, and the variables in the final model yielded parallel curves that did not cross, indicating proportional hazard among the groups. The presence of multicollinearity was checked by using the variance inflation factor (VIF = 1.76), indicating no significant multicollinearity. A Cox proportional hazard regression model was employed to identify the predictors. Variables with a value of p < 0.25 in the bi-variable analysis were candidates for the multivariable Cox proportional hazard model. Hazard ratios (HR) with their respective 95% CI were used to show significance and strength of the association with the outcome variable. Variables having a p-value < 0.05 in the multivariable Cox proportional hazard model were considered significant and independently associated with the outcome variable.

**Fig 1:**
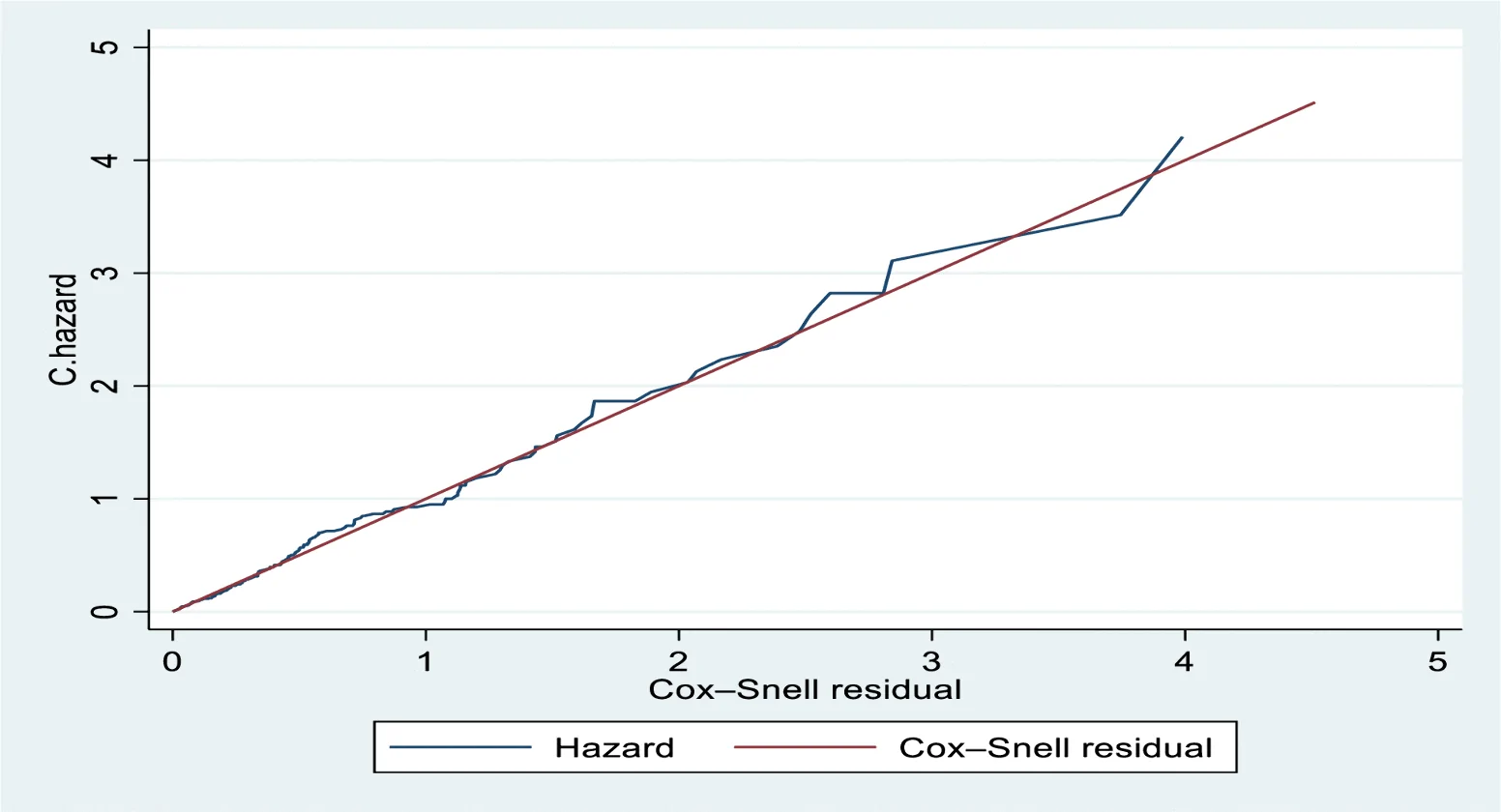
Cumulative hazard vs. Cox–Snell residual plots for Cox regression model of adult patients with diabetes at public hospitals of Harari region, Eastern Ethiopia, 2024. (N = 472).

The overall model fit was evaluated using the Cox-Snell residual plot. The close alignment of the Cox-Snell residual line with the 45-degree cumulative hazard line indicated a well-fitting model, as the residual line closely followed the bisector without significant deviations (Fig 1).

## Results

### Socio-demographic characteristics

From a total of 496 adult diabetes patient cards that were reviewed, 472 were included in the final analysis, while 24 (4.8%) patients with incomplete information and no history of follow-up were excluded. Of the total data, 259 (54.87%) were from HFCSUH and 213 (45.13%) were from Jugal General Hospital. Out of 472 adults with diabetes, half (50.42%) were females, and 257 (54.45%) were urban dwellers. The 25th and 75th percentiles for age at the time of diabetes diagnosis were 34 years and 60 years, respectively, with a median (IQR) age of 46 [26] years. Those who had no health insurance constituted 164(34.75%) of the patients with diabetes (Table 1).

**Table 1:** Incidence of HGE and socio-demographic characteristics of DM patients at public hospitals of Harari region, Eastern Ethiopia, 2024. (N = 472).

Variables	Category	HGE Status		Total (%)
		Developed HGEs(n = 161)	Censored (n = 311)	
Place of follow up	HFCSUH	90	169	259(54.87)
	JGH	71	142	213(45.13)
Age	18-25	40	24	64(13.56)
	26-50	84	138	222(47.03)
	>50	37	149	186(39.41)
Sex	Male	85	149	234(49.58)
	Female	76	162	238(50.42)
Residence	Urban	60	197	257(54.45)
	Rural	101	114	215(45.55)
Community health insurance	Yes	66	242	308(65.25)
	No	95	69	164(34.75)

### Clinical and follow-up characteristics of patients with diabetes

According to this study, more than three-fourths, 361 (76.48%), were patients with type 2 diabetes, and the majority of the patients, 430(91.1%), had no family history of diabetes. Out of the total patients with diabetes, 162 (34.32%) had an acute recent illness. Regarding chronic comorbidities, 264 (55.93%) of patients had comorbidities, and 49 (10.38%) had more than one comorbidity. Cardiovascular disorders 203(43%) were found to be the most common comorbid diseases, of which hypertension accounts for 173(85.22%). Additionally, 208 (44.07%) of patients had poor glycemic control. Overall, 42 patients (8.90%) had recurrent episodes of HGEs. Regarding chronic complications, 36 (7.63%) of patients developed chronic complications of DM. The majority of the patients, 302(63.98%), had a normal body mass index (Table 2).

**Table 2:** Clinical-related results of adult patients with diabetes at public hospitals of Harari region, Eastern Ethiopia, 2024. (N = 472).

Variables	Category	Frequency (%)
Types of DM	Type2DM	361(76.48)
	Type1DM	111(23.52)
DM duration category	<3 years	368(77.97)
	≥ 3 years	104(22.03)
Family history of DM	No	430(91.10)
	Yes	42(8.90)
Recent glycemic control	Good glycemic control	264(55.93)
	Poor glycemic control	208(44.07)
Recent trauma	No	456(96.61)
	Yes	16(3.39)
Body mass index at baseline	Normal	302(63.98)
	Overweight	129(27.33)
	Obesity	27(5.72)
	Underweight	14(2.97)
Acute recent illness (Yes) (No)	Urinary tract infection	64(13.56)
	Respiratory infection	47(9.96)
	Gastrointestinal infection	26(5.51)
	Acute febrile illness	15(3.18)
	Myocardial infarction	11(2.33)
	Others*	17(3.60)
		310(65.68)
Comorbidities (Yes) (No)	Cardiovascular disorder	203(43.00)
	Renal disease	31(6.56)
	Chronic respiratory disorder	19(4.02)
	Liver disease	13(2.75)
	HIV/AIDS	11(2.33)
	Benign prostate hyperplasia	10(2.11)
	Psychiatric disorder	6(1.27)
	Others**	20(4.23)
	More than one comorbidity	49(10.38)
		208(44.07)
Chronic diabetic complication (Yes) (No)	Diabetic neuropathy	13(2.75)
	Diabetic nephropathy	12(2.54
	Diabetic retinopathy	9(1.91)
	Diabetic foot ulcers	4(0.85)
		436(92.37)
Systolic blood pressure (mmHg)	Mean ±(SD) = 135.5 ± 30	
Diastolic blood pressure (mmHg)	Mean±(SD) = 83.6 ± 17	

*Others include carbuncle, cellulitis, pyomyositis, periodontitis, Necrotizing fasciitis, sexually transmitted infection, acute otitis media and osteoarthritis.

**Others include cholelithiasis, goiter, hyperthyroidism, Parkinson disease, epilepsy, hypothyroidism, dyslipidemia, and rectal cancer

### Treatment-related characteristics of adult patients with diabetes

Most of the patients, 330 (69.92%), used oral hypoglycemic agents at diagnosis, and 100 (21.19%) of adult patients with diabetes had medication non-adherence. Almost all, 448(94.92%) of the patients started treatment immediately after diagnosis, and 205(43.43%) patients had monthly follow-up (Table 3).

**Table 3:** Treatment-related results of adult patients with diabetes at public hospitals of Harari region, Eastern Ethiopia, 2024. (N = 472).

Variables	Category	Frequency (%)
Types of drug used at baseline	Oral hypoglycemic agent	330(69.92)
	Insulin	133(28.18)
	Oral and Insulin	9(1.91)
Types of drug used recently	Oral hypoglycemic agent	213(45.13)
	Insulin	172(36.44)
	Oral and Insulin	87(18.43)
Medication non-adherence	No	372(78.81)
	Yes	100(21.19)
Follow up frequency	Regular	309(65.47)
	Irregular	163(34.53)
Duration of treatment started at baseline	Immediate	448(94.92)
	Not immediate	24(5.08)
Urine ketone	<2 mmol/L	356(75.42)
	≥2 mmol/L	116(24.58)
Urine glucose	<2 g/L	334(70.91)
	≥2 g/L	137(29.09)

### Incidence of hyperglycemic emergencies

Among 472 patients with diabetes followed for a period of five years, 161(34.11%), 95% CI (29.96%-38.52%) of them developed HGEs, of which 116(24.58%) were DKA. The incidence rate of HGEs in the cohort during a cumulative 862 person-years of observation (PYO) was 18.6 cases per 100 person-years of observation, 95% CI (15.9-21.7) or 1.5 cases per 100 person-months of observation, 95% CI (1.3-1.7). The incidence rate of DKA was found to be 13.4 cases per 100 person-years (95% CI, 11.2–16.1), which was 32.6 and 8.3 per 100 person-years among T1DM and T2DM, respectively. The incidence rate of HHS was 5.2 cases per 100 person-years (95% CI, 3.8–6.9), which was 3.5 and 5.6 per 100 person-years among T1DM and T2DM, respectively. Throughout the entire follow-up period, a higher incidence of HGEs occurred within the first 0–6 months of follow-up interval (Table 4).

**Table 4:** Life table showing the survival to develop HGEs among adult diabetic patients at public hospitals of Harari region, Eastern Ethiopia, 2024. (N = 472).

Interval in months	Patients at risk	No of HGEs cases	Censored	Cumulative Survival	SD. Error	[95% CI] L U
(0 6]	472	44	29	0.0962	0.0138	0.0725 0.1271
(6 12]	399	34	55	0.1789	0.0184	0.1459 0.2184
(12 18]	310	38	59	0.2901	0.0231	0.2475 0.3383
(18 24]	213	11	24	0.3290	0.0247	0.2833 0.3799
(24 30]	178	15	37	0.3921	0.0272	0.3412 0.4477
(30 36]	126	7	14	0.4278	0.0288	0.3738 0.4863
(36 42]	105	3	10	0.4450	0.0296	0.3893 0.5049
(42 48]	92	6	25	0.4869	0.0319	0.4265 0.5511
(48 54]	61	3	19	0.5168	0.0344	0.4514 0.5856
(54 60]	39	0	39	0.5168	0.0344	0.4514 0.5856

### Survival functions and comparison of various categorical variables

**Fig 2:**
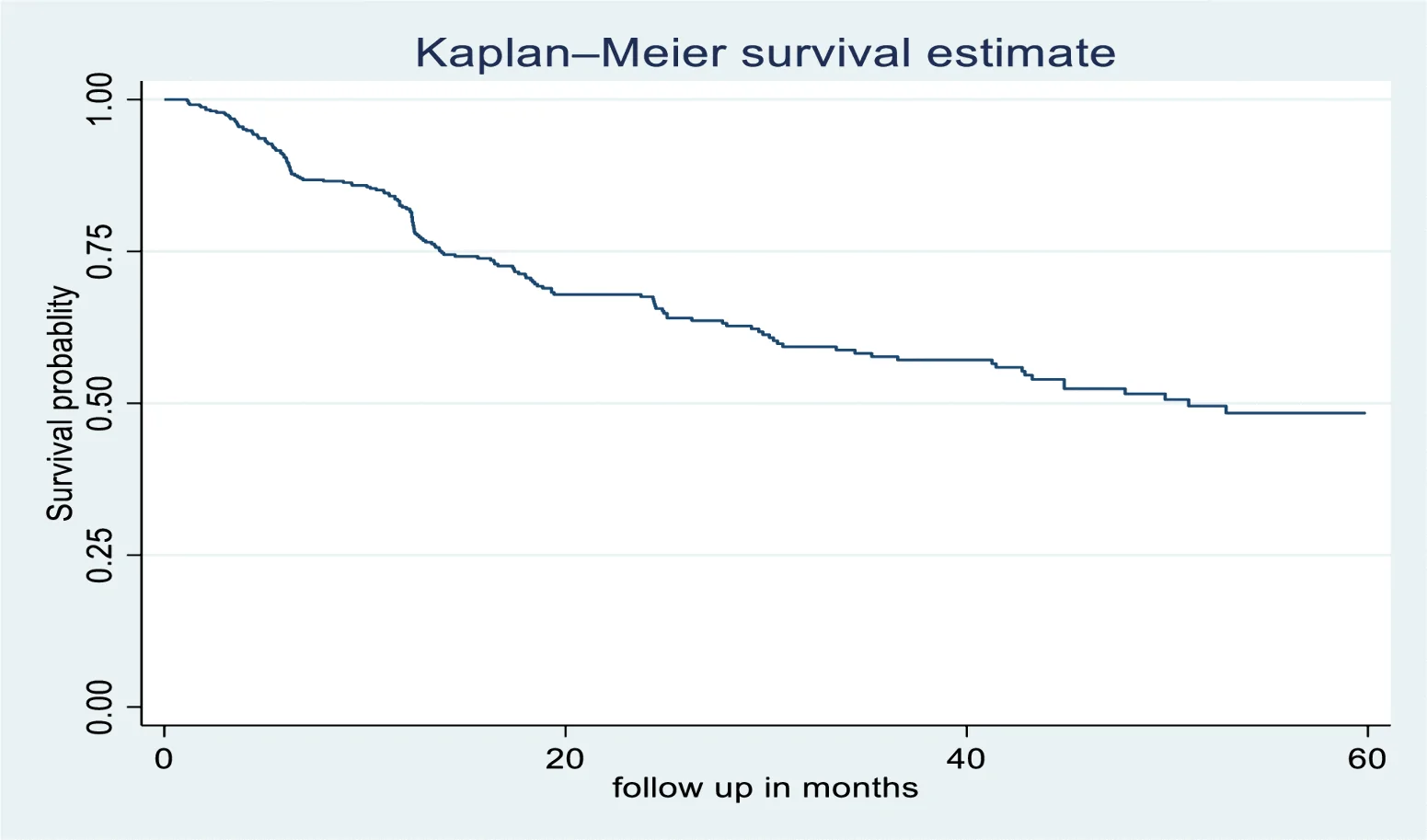
The overall Kaplan–Meier survival curve of HGEs-free survival estimate of adult patients with diabetes at public hospitals of Harari region, Eastern Ethiopia, 2024. (N = 472).

The entire cohort's median HGE-free survival time was 51.06 months (95% CI: 45.14–58.56). The Kaplan–Meier estimate indicated that the probability of HGE-free survival among adult patients with DM was high at 1.13 months of observation (99.79%). However, the probability of HGE-free survival gradually decreased as the follow-up duration increased, reaching a minimum probability of 48.39% at 59.87 months of observation. (Fig 2).

**Fig 3:**
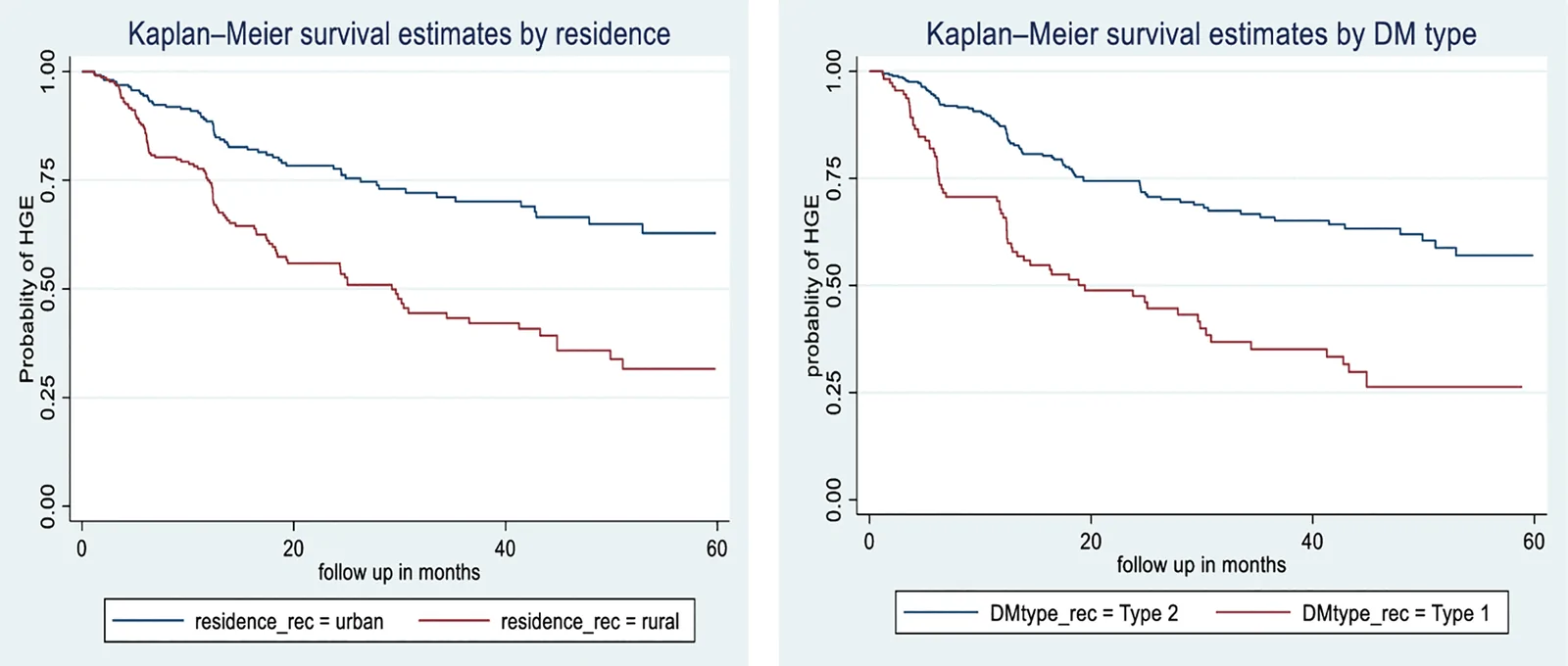
Kaplan-Meier curve for HGE estimates among different groups of diabetic patients by residence and Type of DM of participants at public hospitals of Harari region, Eastern Ethiopia, 2024. (N = 472).

Log-rank test was performed to assess the equality of probability for HGEs and to identify significant differences in survival among different levels of the categorical variables. Patients from urban areas, those with health insurance, individuals with a normal body mass index, patients without a history of medication non-adherence, those with good glycemic control, patients without a history of acute illness, and patients diagnosed with Type 2 DM had longer HGE-free survival time compared to their corresponding categories (Log-rank test, p-value <0.01) (Fig 3).

### Predictors of hyperglycemic emergencies

Variables with a p-value less than 0.25 in the bivariable Cox proportional hazard regression model were selected as candidates for the multivariable Cox regression model to identify predictors of HGEs. A multivariable Cox proportional hazard regression model was fitted with 10 variables, and 7 variables were found to be significant. In the multivariable Cox proportional hazard regression model, residence, community health insurance, acute recent illness, type of DM, recent glycemic control, body mass index, and history of medication non-adherence were significantly associated with HGEs (P < 0.05). Age, follow-up frequency, and the presence of comorbidity were not statistically significant predictors of HGEs.

Accordingly, patients from rural areas had a 52% times higher hazard of developing HGEs than those from urban areas (AHR: 1.52, 95% CI: 1.07, 2.16). Patients without community health insurance had a 63% times higher hazard of developing HGEs than those with community health insurance (AHR: 1.63, 95% CI: 1.14,2.34). Patients who had recently experienced an acute illness were 2.15 times more likely to develop HGEs compared to those who had not (AHR: 2.15, 95% CI: 1.51, 3.07). Adult patients with Type1DM had almost 2 times higher hazard of developing HGEs than adult patients with Type2DM (AHR: 1.97, 95% CI: 1.23, 3.15). Adult patients with diabetes with poor glycemic control had a 3 times higher hazard of developing HGEs compared to patients with good glycemic control (AHR: 3.06, 95% CI: 2.01,4.66).

Regarding BMI, patients with diabetes who were obese and overweight had 3.75 times (AHR: 3.75, 95% CI: 1.84, 7.64) and 2.56 times (AHR: 2.56, 95% CI: 1.72, 3.81) higher hazard of developing HGEs compared to patients with normal BMI, respectively. Furthermore, the hazard of developing HGEs among patients with a history of medication non-adherence was 2.25 times more hazardous relative to those without a history of medication non-adherence (AHR: 2.25, 95% CI: 1.55,3.26) (Table 5).

**Table 5:** Bivariable and Multivariable analysis using Cox proportional hazard regression model for predictors of HGE among DM patients at public hospitals of Harari region, Eastern Ethiopia, 2024. (N = 472).

Variables	Category	HGE status		CHR (95% CI)	AHR (95% CI)	P-value
		Developed HGEs (%)	Censored (%)			
Age	18-25	40(62.5)	24(37.5)	1	1	1
	26-50	84(37.8)	138(62.2)	0.50(0.34,0.73)	1.33(0.79,2.23)	0.274
	>50	37(19.9)	149(80.1)	0.26(0.17,0.41)	0.88(0.46,1.69)	0.708
Residence	Urban	60(23.3)	197(76.7)	1	1	1
	Rural	101(47.0)	114(53.0)	2.39(1.73,3.29)	1.52(1.07,2.16)	**0.019**
Community health insurance	Yes	66(21.4)	242(78.6)	1	1	1
	No	95(57.9)	69(42.1)	3.30(2.41,4.52)	1.63(1.14,2.34)	**0.007**
Types of DM	Type2DM	94(26.0)	267(74.0)	1	1	1
	Type1DM	67(60.4)	44(39.6)	2.61(1.91,3.58)	1.97(1.23,3.15)	**0.004**
Recent glycemic control	GGC	41(15.5)	223(84.5)	1	1	1
	PGC	120(57.7)	88(42.3)	4.84(3.39,6.91)	3.06(2.01,4.66)	**<0.001**
BMI	normal	71(23.5)	231(76.5)	1	1	1
	underweight	8(57.1)	6(42.9)	2.70(1.30,5.62)	1.55(0.65,3.66)	0.314
	overweight	72(55.8)	57(44.2)	2.69(1.93,3.74)	2.56(1.72,3.81)	**<0.001**
	obesity	10(37.0)	17(63.0)	1.96(1.01,3.80)	3.75(1.84,7.64)	**<0.001**
Acute recent illness	No	64(20.6)	246(79.4)	1	1	1
	Yes	97(59.9)	65(40.1)	3.59(2.62,4.94)	2.15(1.51,3.07)	**<0.001**
Comorbidities	No	50(24.0)	158(76.0)	1	1	1
	Yes	111(42.0)	153(58.0)	1.85(1.32,2.59)	1.28(0.89,1.84)	0.175
Follow up frequency	Regular	78(21.6)	231(78.4)	1	1	1
	Irregular	83(50.9)	80(49.1)	0.72(0.49,1.05)	0.70(0.44,1.10)	0.129
Medication non-adherence	No	79(21.2)	293(78.8)	1	1	1
	Yes	82(82.0)	18(18.0)	2.56(1.80,3.63)	2.25(1.55,3.26)	**<0.001**

CHR, crude hazard rate; AHR, adjusted hazard rate; 1 shows a reference category; PGC, poor glycemic control; GGC, good glycemic control; BMI, body mass index.

## Discussion

In this study, the incidence of HGEs was high. Rural residence, lack of community health insurance, acute recent illness, Type1DM, poor glycemic control, history of medication non-adherence, overweight, and obesity were found to be significantly associated with the hazard of HGEs.

The incidence rate of HGEs in this study was higher than the study in Bahirdar, Ethiopia (14.6 per 100 PY) [17], Spain(2.9 per 1000 PY) [34], and Korea (2.15 per 1000 PY) [35]. The incidence rate of DKA was 13.4 per 100 person-years. This is higher than a study conducted in Bahirdar, Ethiopia (12.5 per 100 PY) [17] and a systematic study conducted in different parts of the world (0–56 per 1000 PY). However, this is lower than in China (263 per 1000 PY) [15]. The incidence rate of DKA among T1DM and T2DM is in line with a study conducted in Bahirdar, Ethiopia (35.6 and 6.3 per 100 PY among T1DM and T2DM, respectively) [17], but higher than a study conducted in Western Australia (178.6 per 10000 and 13.3 per 10000 person-years for type 1 and type 2 diabetes, respectively) [36]. This variation may be attributed to differences in health infrastructure, treatment protocol, socioeconomic status, and lifestyle factors, which could have contributed to the increased incidence of DKA [32,37].

The incidence rate of HHS among T1DM and T2DM in this study is much higher than the national reports from the United States (0.9 per 1000 PY) [10] and Denmark(20.4 per 10,000 PY (16.5 and 3.9 among people with known type 1 and type 2 DM, respectively) [38]. The higher incidence rates of DKA and HHS may be explained by the institution-based nature of the current study, which likely captured more severe or advanced cases compared to broader, population-based national studies. Another possible explanation could be differences in economic status, educational attainment, and access to healthcare services between the populations studied previously and the population in our study.

The median hyperglycemic emergency-free survival time was 51.06 months, which is shorter than the study in Bahirdar, Ethiopia [39]. This difference might be attributed to several factors, including variations in patient demographics, access to healthcare, and differences in the management of diabetes.

The higher incidence of HGEs observed among patients residing in rural areas and those without community health insurance underscores the critical role of healthcare accessibility and affordability. These factors contribute to poor diabetes management and increase the risk of HGEs [40–42]. Similar associations have been reported in studies conducted in Bahirdar, Ethiopia [17], Iraq [5] and the USA [7], suggesting that structural barriers to care remain a key determinant of acute diabetes complications.

This study found a strong association between poor glycemic control and the risk of HGEs. This finding aligns with other studies done in different parts of Ethiopia; at Jimma Medical Center [28], Bahir Dar public hospitals [17], Gurage Zone Hospitals [27], and also this study is comparable with a study conducted in Western Australia [36]. The likely explanation is that persistent hyperglycemia, particularly due to insulin deficiency, triggers the release of counter-regulatory hormones such as glucagon, cortisol, and catecholamines. These hormones increase glucose production and promote fat breakdown, leading to ketone accumulation and the development of diabetic ketoacidosis (DKA). Although hypoglycemia does not directly cause HGEs, it can contribute indirectly through overcorrection, skipped insulin doses, and hormonal rebound responses [43,44]. Patients who had recently experienced an acute illness were more likely to develop HGEs in our study. This finding is consistent with other research from Ethiopia [17,26,28], Cameroon [45], and Iraq [5]. The likely reason for this association is that acute illness often triggers a state of increased metabolism, leading to the release of cytokines and counter-regulatory hormones [43]. This metabolic shift can lead to insulin resistance and enhanced hepatic glucose production, ultimately contributing to the development of HGEs [46].

This study revealed that a history of medication non-adherence is a significant predictor of HGEs. This finding aligns with previous research from Ethiopia [17,30] and Cameroon [45], suggesting a consistent link between medication non-adherence and increased risk of HGEs. This increased risk is likely due to the direct impact of non-adherence on blood glucose levels, as inadequate antidiabetic medication management can lead to uncontrolled hyperglycemia, a primary cause of HGEs(43). The strong association between T1DM and HGEs observed in our cohort reflects established pathophysiological mechanisms. This finding is supported by another study conducted in Ethiopia [17,28,30] and Canada [47]. A plausible reason for this is that DKA is the more frequent hyperglycemic emergency and commonly occurs in T1DM, whereby in T1DM, insulin secretion is absent, hence the higher likelihood of DKA occurring [43].

Regarding body mass index, in this study, patients with diabetes who were obese and overweight were more likely to develop HGE. This is supported by studies conducted in South West Ethiopia [27] and Western Australia [36]. This may be because an increased BMI can lead to acute complications of diabetes mellitus primarily through insulin resistance, which impairs blood glucose regulation and increases the risk of conditions such as DKA and HHS. Obesity is often associated with low-grade inflammation, contributing to metabolic dysregulation and exacerbating insulin resistance. Furthermore, elevated fat deposition, particularly visceral fat, disrupts normal metabolic processes, leading to blood glucose fluctuations [46,47].

Furthermore, while some studies have suggested an association of age [5,25,48–50], sex [5,47,48], diabetes duration [5,17], presence of comorbidities [17,28,30,51], and follow-up frequency [17,30] with HGEs, other studies have found no such association. This study, which also found no significant association between these factors and HGEs, suggests that the inconsistencies across different studies might be due to variations in research methods, cultural contexts, diagnosis, and treatment modalities. Additionally, because the data was collected retrospectively, there may be some missing or incomplete information. These findings suggest the need for further research to better understand the predictors of HGEs.

### Limitations of the study

The retrospective nature of the study limited the inclusion of all potential factors that could affect the HGE status of the patients. Some potentially important predictors, such as educational status, occupation, substance use, and self-care practices, were not available, which prevents us from studying their association with HGE. The study relied on physicians’ judgments to define and diagnose HGE, which may not be sufficiently reliable on its own. Furthermore, the study did not differentiate the specific predictors of the types of HGEs. This limits the ability to conclude about specific predictors for each type of HGEs. Additionally, there remains the possibility of misclassification and documentation bias. Finally, the generalizability of our findings may be limited as the study population was restricted to patients attending public hospitals in the region. However, it is important to note that public hospitals are the primary source of diabetes care for the vast majority of patients in this region, due to their accessibility, affordability, and established chronic care programs. While a small proportion of individuals may receive care from private health facilities, health centers, or clinics, these settings were not included in this study.

## Conclusion and recommendation

In this study, the incidence of HGEs was high. The median HGE-free survival time was 51.06months. Rural residence, lack of community health insurance, acute recent illness, Type1DM, poor glycemic control, history of medication non-adherence, overweight and obesity were found to be significantly associated with the hazard of HGEs. We recommend that special emphasis be given to follow-up care for diabetic patients with identified predictors to reduce the occurrence of HGEs. Finally, we suggest further prospective follow-up studies to address missed variables and to focus on specific HGE types in order to identify specific predictors and develop targeted interventions for each type.

## Supporting information

S1 Data This is the data set of a study of Incidence and Risk Factors of Hyperglycemic Emergencies in Adults with Diabetes. (XLS)

S2 Data This is the checklist of a study of Incidence and Risk Factors of Hyperglycemic Emergencies in Adults with Diabetes. (DOCX)

## Abbreviations

AHR: Adjusted Hazard Ratio
AOR: Adjusted Odd Ratio
ADA: American Diabetes Association
BMI: Body Mass Index
DBP: Diastolic Blood Pressure
DM: Diabetes Mellitus
DKA: Diabetic Ketoacidosis
FBS: Fasting Blood Sugar
GGC: Good Glycemic Control
HbA1C: Hemoglobin A1C or Glycated Hemoglobin
HFCSUH: Hiwot Fana Comprehensive Specialized University Hospital
HGEs: Hyperglycemic Emergencies
HHS: Hyperglycemic Hyperosmolar Syndrome/State
IRERC: Institutional Health Research Ethics Review Committee
IDF: International Diabetes Federation
PGL: Poor Glycemic Control
PM: Person Months
PY: Person Years
RBGL: Random Blood Glucose Level
SBP: Systolic Blood Pressure
T1 DM: Type 1 Diabetes Mellitus
T2 DM: Type 2 Diabetes Mellitus
WHO: World Health Organization
