# Supplementary material for: Incidence and risk factors of hyperglycemic emergencies in adults with diabetes in the public hospitals in Harari region, Eastern Ethiopia
Source: PLOS Glob Public Health. 2026 Sep 25;6(9):e0007345. doi: 10.1371/journal.pgph.0007345 (PMC13614554; doi:10.1371/journal.pgph.0007345)
Supplement: Supplementary file 1 [file pgph.0007345.s002.docx]

## **Data Extraction Check List**

**Topic:** Incidence and risk factors of hyperglycemic emergencies among adult diabetes mellitus patients at public hospitals of Harari Region, Eastern Ethiopia.

**Instruction**: Dear data collector this is a data abstraction form designed to collect data of patients started the treatment in Public Hospitals of Harari region. Please try to fill all the requested fields that are prepared in the section of the data abstraction form.

**The form has five sections:**

The First section: General information

The Second section: The Socio-demographic characteristics of the patient

The Third section: The baseline clinical characteristics

The Fourth section: Treatment related factors

The Fifth section: Follow up form

**Part I: General information**

**G001**. Date: ___________________________________

**G002**. Name of the health facility ___________________

**G003**. Code given for the chart by investigator_______________________

**G004**. Name of the data collector________________________

**G005**. Name of the supervisor________________________

**Part II: Baseline Socio-demographic characteristics of DM patients**

| **Code** | **Characteristics** | **Coding classification** |
| --- | --- | --- |
| **B101** | Sex | 1. Male  2. Female |
| **B102** | Age | _______Years. |
| **B103** | Place of residence | 1. Urban  2. Rural |
| **B104** | Community health insurance | 0. Yes  1. No |

**Part III: Baseline Clinical Characteristics of DM Patients**

| **C108** | Date the patient confirmed as a Diabetic patient | dd/mm/yy_____/_____/____ |
| --- | --- | --- |
| **C109** | Family history of DM | 0. No  1. Yes |
| **C110** | Types of DM | 1. Type 1  2. Type 2 |
| **C111** | Trauma | 0. No  1. Yes |
|  | **Acute recent illness** |  |
| **C111** | Acute febrile illness  If yes specify | 0. No  1. Yes |
| **C112** | Respiratory infection  If yes specify | 0. No  1. Yes |
| **C113** | Urinary tract infection | 0. No  1. Yes |
| **C114** | Gastrointestinal infection  If yes specify | 0. No  1. Yes |
| **C115** | Myocardial infarction | 0. No  1. Yes |
| **C116** | Other acute recent illness | ______________________ |
|  | **Comorbidities** |  |
| **C117** | Chronic respiratory disorder  If yes specify | 0. No  1. Yes |
| **C118** | Cardiovascular disorder  If yes specify | 0. No  1. Yes |
| **C119** | HIV/AIDS | 0. No  1. Yes |
| **C120** | Psychiatric disorder | 0. No  1. Yes |
| **C121** | Renal disease  If yes specify | 0. No  1. Yes |
| **C122** | Liver disease  If yes specify | 0. No  1. Yes |
| **C124** | Another comorbidity | ______________________ |
| **C125** | Recent glycemic control | 1. Good glycemic control  2. Poor glycemic control |
|  | **Complication of DM** |  |
| **C126** | Acute Complication of DM | 0. No  1. Yes |
| **C127** | If present which Acute complication  of DM | 1. DKA  2. HHS  3. Hypoglycemia |
| **C128** | If DKA present how many episode | 1. One  2. Two  3. Three  4. Four  5. Five or more |
| **C129** | If HHS present how many episode | 1. One  2. Two  3. Three  4. Four  5. Five or more |
| **C130** | Chronic complication | 0. No  1. Yes |
| **C131** | If present which chronic complication  of DM | 1. Diabetic retinopathy  2. Diabetic foot ulcers  3. Diabetic neuropathy  4. Diabetic nephropathy  5. others(list)____________ |
| **C132** | BMI at diagnosis | Weight _________  Height _________ |

**Part IV: Treatment-related factors**

| **T133** | Types of medication used at baseline | 1. Oral hypoglycemic agent  2. Insulin  3. Oral and Insulin |
| --- | --- | --- |
| **T134** | Types of medication used recently | 1. Oral hypoglycemic agent  2. Insulin  3. Oral and Insulin |
| **T135** | Medication non-compliance | 0. No  1. Yes |
| **T136** | Follow up frequency | 1. Weekly  2. Monthly  3. 2-3 months  4. ≥4 months  5. Irregular |
| **T137** | Duration of treatment started | 1. Immediate  2. Not immediate |

**Part V: Follow up form to be filled**

| Follow up time  (in months) | **HGEs status** | | | | | | Date | FBS | Urine ketone | Urine  glucose | SBP | DBP |
| --- | --- | --- | --- | --- | --- | --- | --- | --- | --- | --- | --- | --- |
|  | DKA | HHS | LTF | TO | Died | Still not developed |  |  |  |  |  |  |
|  |  |  |  |  |  |  |  |  |  |  |  |  |
| At diagnosis |  |  |  |  |  |  |  |  |  |  |  |  |
| 6 |  |  |  |  |  |  |  |  |  |  |  |  |
| 12 |  |  |  |  |  |  |  |  |  |  |  |  |
| 18 |  |  |  |  |  |  |  |  |  |  |  |  |
| 24 |  |  |  |  |  |  |  |  |  |  |  |  |
| 30 |  |  |  |  |  |  |  |  |  |  |  |  |
| 36 |  |  |  |  |  |  |  |  |  |  |  |  |
| 42 |  |  |  |  |  |  |  |  |  |  |  |  |
| 48 |  |  |  |  |  |  |  |  |  |  |  |  |
| 54 |  |  |  |  |  |  |  |  |  |  |  |  |
| 60 |  |  |  |  |  |  |  |  |  |  |  |  |
